# A three-country analysis of the gut microbiome indicates taxon associations with diet vary by taxon resolution and population

**DOI:** 10.1128/msystems.00544-25

**Published:** 2025-06-30

**Authors:** Lora Khatib, Se Jin Song, Amanda H. Dilmore, Jon G. Sanders, Caitriona Brennan, Alejandra Rios Hernandez, Tyler Myers, Renee Oles, Sawyer Farmer, Charles Cowart, Amanda Birmingham, Edgar A. Diaz, Oliver Nizet, Kat Gilbert, Nicole Litwin, Promi Das, Brent Nowinski, Mackenzie Bryant, Caitlin Tribelhorn, Karenina Sanders-Bodai, Soline Chaumont, Jan Knol, Guus Roeselers, Manolo Laiola, Sudarshan A. Shetty, Patrick Veiga, Julien Tap, Muriel Derrien, Hana Koutnikova, Aurélie Cotillard, Christophe Lay, Armando R. Tovar, Nimbe Torres, Liliana Arteaga, Antonio González, Daniel McDonald, Andrew Bartko, Rob Knight

**Affiliations:** 1 Department of Pediatrics, University of California San Diego 547075, La Jolla, California, USA; 2 Neurosciences Graduate Program, University of California San Diego 8784, La Jolla, California, USA; 3 Center for Microbiome Innovation, University of California San Diego 8784, La Jolla, California, USA; 4 Biomedical Sciences Program, University of California San Diego 8784, La Jolla, California, USA; 5 Division of Biological Sciences, University of California San Diego 8784, La Jolla, California, USA; 6 Department of Chemical and Biomolecular Engineering, Johns Hopkins University 198421, Baltimore, Maryland, USA; 7 Crohn’s and Colitis Foundation, New York, New York, USA; 8 Danone Research and Innovation, Gif sur Yvette, France; 9 Danone Research and Innovation, Utrecht, the Netherlands; 10 Laboratory of Microbiology, Wageningen University and Research 568404, Wageningen, the Netherlands; 11 Precision Nutrition D-Lab, Danone Global Research and Innovation Center, Singapore, Singapore; 12 Instituto Nacional de Ciencias Médicas y Nutrición Salvador Zubirán, Mexico City, México; 13 Department of Bioengineering, University of California San Diego 8784, La Jolla, California, USA; 14 Department of Computer Science and Engineering, University of California San Diego 214553, La Jolla, California, USA; Pontificia Universidad Catolica de Chile, Santiago, Chile; Universidad de Leon, Leon, Spain; University of Groningen, Groningen, the Netherlands

**Keywords:** human microbiome, metagenomics, diet, *Prevotella*, *Faecalibacterium*

## Abstract

Emerging research suggests that diet plays a vital role in shaping the composition and function of the gut microbiota. Although substantial efforts have been made to identify general patterns linking diet to the gut microbiome, much of this research has been concentrated on a small number of countries. Additionally, both diet and the gut microbiome have highly complex and individualized configurations, and there is growing evidence that tailoring diets to individual gut microbiota profiles may optimize the path toward improving or maintaining health and preventing disease. Using fecal metagenomic data from 1,177 individuals across three countries, we examine the relationship between diet and bacterial genera, focusing on *Prevotella* and *Faecalibacterium*, which have gained significant attention for their potential roles in human health and strong associations with dietary patterns. We find that these two genera in particular show significant associations with many aspects of diet but these associations vary in scale and direction, depending on the level of metagenomic resolution (i.e., genus level by reads and strain level by metagenome-assembled genomes) and the contextual population. These results highlight the growing importance of building metagenomic data sets that are standardized, comprehensive, and representative of diverse populations to increase our ability to tease apart the complex relationship between diet and the microbiome.

**IMPORTANCE:**

An analysis of fecal microbiome data from individuals in the United States, United Kingdom, and Mexico shows that associations with dietary components vary both by country and by level of resolution (i.e., genus and strain). Our work sheds light on why there may be conflicting reports regarding microbial associations with diet, disease, and health.

## OBSERVATION

**Fig 1:**
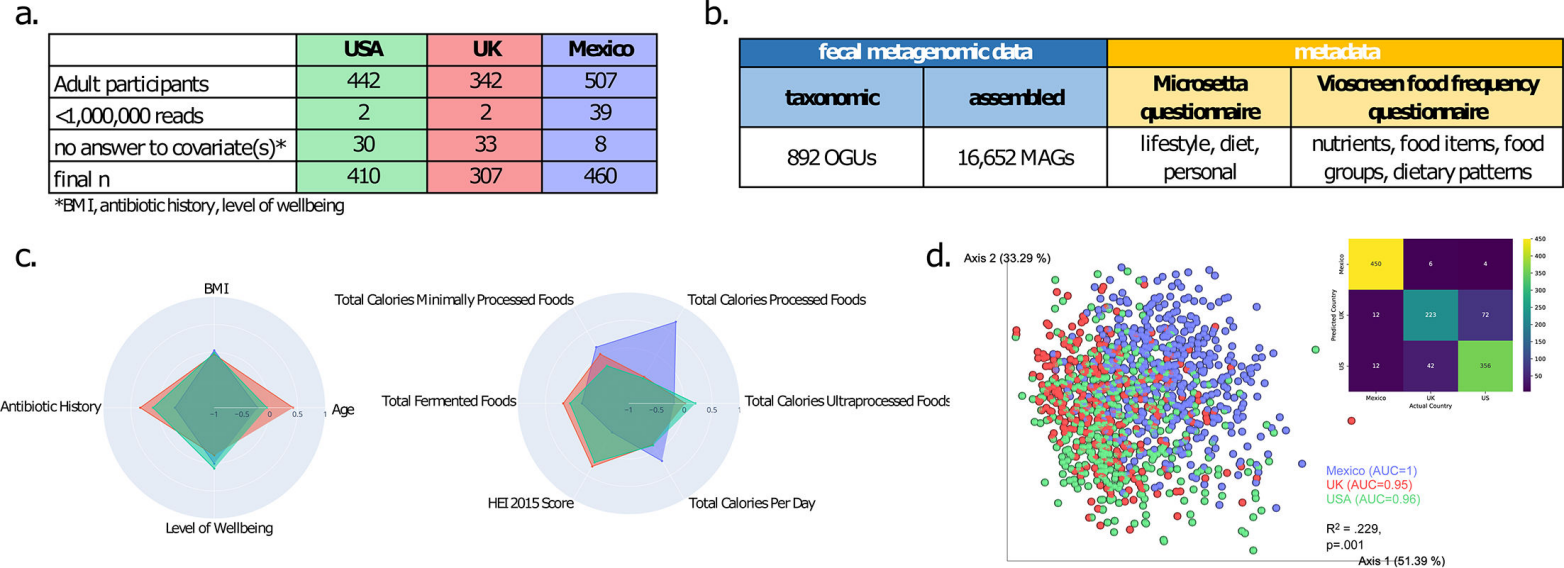
Study and data overview. (**a**) The table shows the number of samples analyzed by shotgun metagenomics from each country and the resulting sample size after filtering steps. (**b**) Data collected included taxonomic profiles and MAGs from the sequence data, and diet and lifestyle information from self-reported answers to questionnaires. (**c**) Radar plots show *z*-normalized values for key personal and diet-related variables, averaged by country. (**d**) A principal coordinates analysis plot shows robust Principal Component Analysis (PCA) distance among the microbiome samples, colored by the cohort country. Partial R2 from a Permutational Multivariate Analysis of Variance (PERMANOVA) is reported. A confusion matrix shows the classification accuracy using a random forest classifier (fivefold cross validation) on the taxonomic feature table. The mean Area Under the Curve (AUC) for the Receiver Operating Characteristic (ROC) curve is also reported.

In this study, we explored the relationships between the gut microbiome at different levels (taxonomic genera and metagenome-assembled genomes [MAGs]) and various dietary factors using data collected from subjects in three countries (Fig. 1a and b). We conducted metagenomic sequencing of fecal samples from 442 adult participants in the United States (US), 342 in the United Kingdom (UK), and 507 in Mexico, recruited through the Microsetta Initiative platform (1). We evaluated long-term dietary intake using the VioScreen food frequency questionnaire (FFQ; Version 5, VioCare, Princeton, NJ) (2), adapting it for Mexico to include common regional foods (Version 5-Mex). Consumption of food groups (kcal/day) was derived from raw VioScreen entries, applying several filtering criteria to ensure data quality based on the number of items consumed, total energy intake, and FFQ completion time as previously described (3).

All cohorts included more females than males, with an average body mass index (BMI) of 25 (±5 SD), but varied in other characteristics. Subjects in the UK were older, while those in Mexico reported higher incidences of diabetes and irritable bowel disease (IBD) and the lowest diet quality (Healthy Eating Index [HEI]; Fig. 1c; Table 1). However, all three cohorts constituted individuals healthier than the general US population, reporting a lower BMI, higher HEI, and lower incidence of diabetes and cardiovascular disease (4, 5).

**TABLE 1:** Key demographic, health, and diet characteristics of the three cohorts

Parameter	Mexico	UK	US	Statistic	*P*-value
No. of participants	460 (39.1%)	307 (26.1%)	410 (34.8%)	31.026	1.83e−07
Age	42.195 ± 15.133	52.116 ± 13.269	45.011 ± 15.22	81.618	1.89e−18
Sex: female	307 (66.7%)	223 (72.6%)	262 (63.9%)	6.191	0.04525496
Education level: college graduate	206 (45.8%)	153 (50.2%)	189 (46.8%)	20.238	0.00044809
Alcohol frequency: regularly	36 (7.9%)	80 (26.4%)	92 (22.4%)	52.158	4.72e−12
BMI	25.573 ± 5.01	25.091 ± 5.706	25.23 ± 5.314	5.114	0.0775445
Diabetes	31 (6.9%)	9 (3.0%)	15 (3.7%)	7.777	0.02047483
Cardiovascular disease (CVD)	9 (2.0%)	14 (4.6%)	16 (3.9%)	9.818	0.04360907
Autoimmune disease	19 (4.3%)	51 (17.0%)	52 (12.8%)	34.029	4.08e−08
Inflammatory bowel disease (IBD)	64 (15.1%)	9 (3.0%)	9 (2.3%)	61.016	5.63e−14
Irritable bowel syndrome (IBS)	123 (28.4%)	82 (27.1%)	72 (18.3%)	12.9	0.00158089
Gluten intolerance	26 (6.3%)	31 (10.3%)	51 (12.9%)	23.303	0.00070106
Lactose intolerance	136 (31.1%)	29 (9.9%)	74 (18.6%)	49.665	1.64e−11
Bowel movement, normal	349 (77.0%)	218 (73.4%)	300 (76.5%)	30.367	3.35e−05
Diet type (vegetarian)	6 (1.3%)	21 (6.9%)	27 (6.6%)	83.655	8.96e−15
Plant diversity (more than 20)	55 (12.1%)	105 (34.9%)	96 (23.5%)	189.206	1.20e−36
Vegetable frequency, regularly	358 (78.0%)	286 (93.5%)	345 (84.8%)	37.794	1.24e−07
Fruit frequency, regularly	314 (68.6%)	207 (67.9%)	251 (61.5%)	6.348	0.17461969
Whole grain frequency, regularly	194 (42.7%)	155 (50.7%)	243 (59.7%)	25.219	4.55e−05
Red meat frequency, regularly	171 (37.6%)	46 (15.0%)	60 (14.7%)	90.161	1.22e−18
Milk/cheese frequency, regularly	303 (66.0%)	188 (61.2%)	198 (48.9%)	28.107	1.19e−05
Sugar-sweetened beverage frequency, regularly	46 (10.1%)	7 (2.3%)	13 (3.2%)	76.907	7.87e−16
Energy intake from the diet (kcal/day)	2284.797 ± 901.074	2007.523 ± 688.092	2017.892 ± 678.689	21.896	1.76e−05
Ultraprocessed foods (kcal/day)	345.554 ± 127.168	438.919 ± 141.507	487.323 ± 152.632	195.972	2.79e−43
Processed foods (kcal/day)	213.998 ± 100.985	80.455 ± 62.177	77.713 ± 59.797	493.984	5.40e−108
Minimally processed foods (kcal/day)	388.19 ± 121.734	411.377 ± 130.741	359.726 ± 133.215	34.562	3.13e−08
Carbohydrate (%)	47.283 ± 7.625	46.105 ± 9.872	44.178 ± 9.259	28.152	7.71e−07
Fat (%)	34.088 ± 6.295	34.988 ± 8.698	36.854 ± 8.159	30.223	2.74e−07
Protein (%)	16.307 ± 3.189	14.753 ± 3.007	15.341 ± 3.526	47.901	3.97e−11
HEI (2010)	64.741 ± 9.495	73.197 ± 10.537	72.456 ± 10.516	182.008	3.00e−40
HEI (2015)	63.348 ± 9.308	71.027 ± 10.242	70.166 ± 10.398	147.01	1.19e−32

Stool collection and DNA extraction were performed similarly across the three cohorts. Sample libraries generated an average of 4,559,420 sequences per sample (±1,750,993 SD), which were quality controlled and human sequence filtered following Armstrong et al. (6), before generating operational genomic units through the woltka pipeline (7) (Fig. 1b). A robust PCA analysis (8) showed that geographic location had the strongest effect on microbiome composition (partial *R*^2^ = 0.229, *P* = 0.001; Fig. 1d), enabling a random forest classifier to predict location with high accuracy (mean AUC: 1.00 for Mexico, 0.95 for UK, and 0.96 for USA) across fivefold cross-validation. The next top variables showing significant effects not related to diet were antibiotic history (*R*^2^ = 0.0137, *P* = 0.001), mental and physical well-being (*R*^2^ = 0.0132, *P* = 0.001), and BMI (*R*^2^ = 0.0076, *P* = 0.001). Therefore, all downstream analyses, where possible, included these factors as covariates.

**Fig 2:**
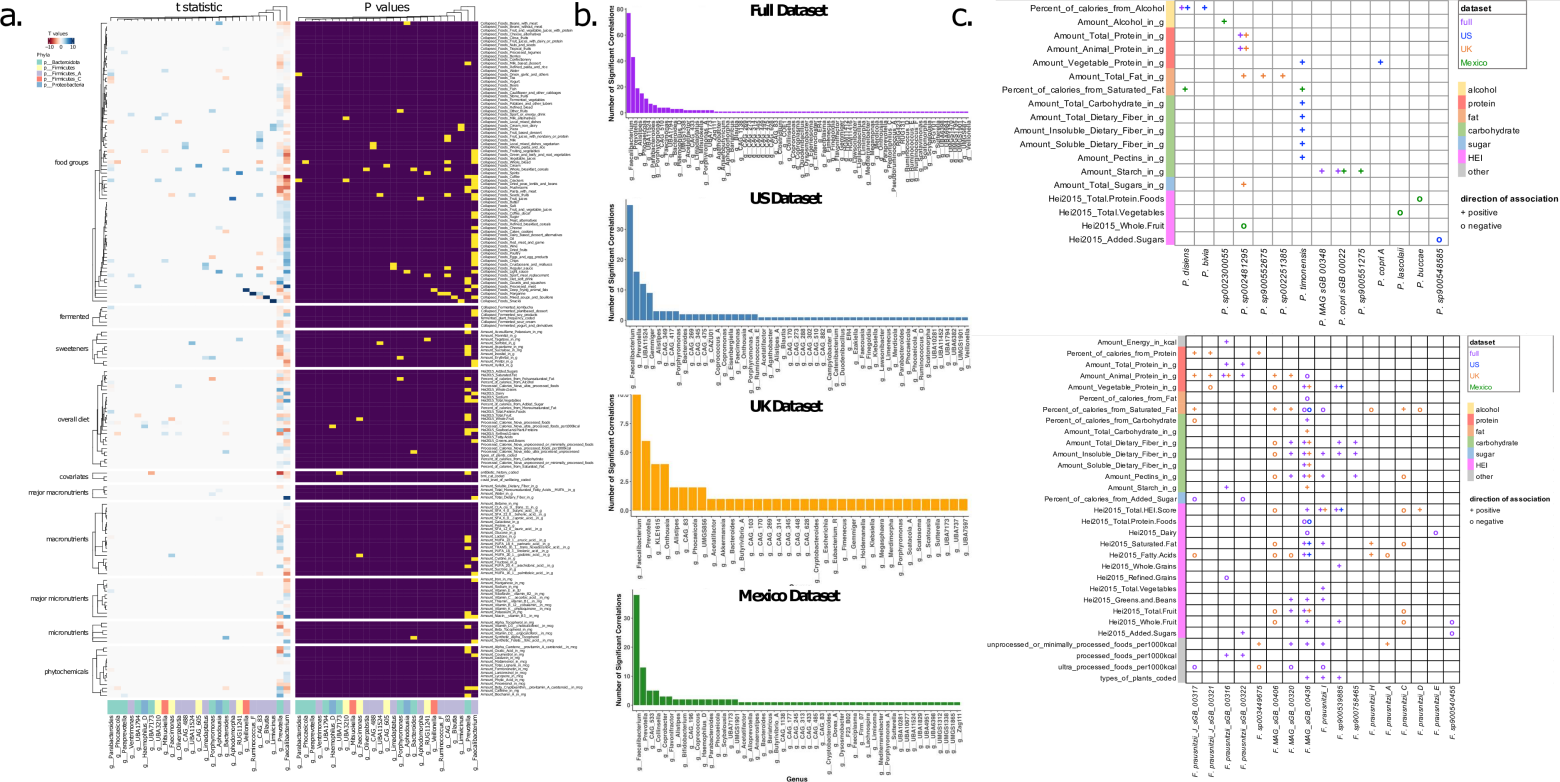
Aspects of *Prevotella* and *Faecalibacterium* at different levels of resolution highlight the complexity of diet-microbe associations, each showing ties with many dietary variables, but varying in direction and strength by country. (**a**) Heatmap shows *t*-scores and *P*-values for testing the correlation between the nucleotide diversity in sGBs and dietary variables, pooled per individual by bacterial genus. (**b**) Bar plots show the number of significant correlations between each bacterial genus and dietary variables across all participants (purple), as well as within each country: US (blue), UK (orange), and Mexico (green). (**c**) An association map shows the dietary variables that were significantly correlated with the log ratio of each *Prevotella* sGB to the sum of all *Prevotella* (top) and each *Faecalibacterium* sGB to the sum of all *Faecalibacterium* (bottom) in the full data set and stratified by country, after accounting for the variation explained by covariates (cohort, BMI, antibiotic history, and level of well-being). Multiple comparisons were corrected using the Benjamini-Hochberg method with a 5% False Discovery Rate (FDR).

To narrow the focus for downstream, strain-resolved analyses, we assembled contigs and derived species-level genome bins (sGBs) using dRep (9), following Sanders et al. (10). Next, we calculated nucleotide diversity within each sGB for each individual using InStrain (11) and then down-selected from 286 dietary variables using linear LASSO regression, followed by linear mixed-effects models. This investigation revealed that *Prevotella* (including some species now reclassified under different genera, e.g., *Segatella copri*) and *Faecalibacterium* nucleotide diversity showed the strongest associations with diet (Fig. 2a). This finding was consistent across countries (Fig. 2b) but varied across certain covariate subgroups—though *Prevotella* and *Faecalibacterium* consistently showed more associations than most genera (Fig. S1). *Prevotella* diversity across all participants was positively associated with whole grain consumption (*t* = 4.962, *P* = 7.21e−7) and negatively with antibiotic use (*t* = −6.949, *P* = 4.17e−12). *Faecalibacterium* diversity was positively associated with dietary fiber (*t* = 9.282, *P* = 2.20e−20), consistent with previous studies (12), and, unexpectedly, with processed meat (*t* = 9.060, *P* = 1.67e−19)—possibly reflecting participants in Mexico who reported high fiber and processed meat consumption.

Next, we assessed the associations between the strain-level abundance of these two genera and a subset of dietary variables that broadly captured overall dietary patterns (Table S1). Only 14 out of 56 *Prevotella* sGBs showed significant associations with dietary variables (Fig. 2c). Few *Prevotella* sGBs showed consistency across the cohorts. In the full data set, *Prevotella disiens* was positively associated with alcohol consumption, *P. sp002481295* was associated with animal and protein intake, and two sGBs, *P. MAG sGB 00348* and *P. copri sGB 00022* (i.e*., Segatella copri*), were associated with starch intake. However, the remaining 10 sGBs showed associations that were country specific. For example, *P. timonensis* (i.e., *Hoylesella timonensis*) showed positive associations with vegetable and fiber intake in the US, but not in the UK or Mexico, where it was instead associated with saturated fat intake.

In contrast, 17 out of 19 of the *Faecalibacterium* sGBs showed significant associations (Fig. 2c). Several *Faecalibacterium* sGBs (most notably *F. MAG_sGB_00436*, *F. prausnitzii_I*, and *F. sp900539885*) were positively associated with intake of vegetable, fruit, carbohydrates (including fibers, pectin, and starch), and unprocessed or minimally processed foods, as well as an overall healthy diet (high HEI), while negatively associated with animal protein and dairy. However, other sGBs (*F. prausnitzii_C* and *F. MAG_sGB_00406*) showed opposite trends, being associated with animal protein, saturated fat, and an overall poorer diet (low HEI), but only in the UK cohort. Interestingly, no sGBs were found to be significantly associated with any dietary variables in Mexico.

Lastly, we examined whether the Centered Log-Ratio (CLR)-transformed values of *Prevotella* and *Faecalibacterium* displayed a similar pattern to nucleotide diversity. We found that, when analyzed on the genus level, only *Prevotella* exhibited a positive correlation with starch intake (*R*^2^ = 0.0317, *P* = 0.04), and this association was only consistent in participants from Mexico (Fig. S2a). The absence of consistent genus-level abundance associations, in contrast to diversity and strain-level findings, emphasizes that resolving microbiome features to the strain level is essential for identifying biologically meaningful relationships with diet.

Understanding the complex associations between diet and the microbiome can contribute to our knowledge of gut microbiota modulation and its implications for personalized nutrition. Here, we focused on two bacterial genera, *Prevotella* and *Faecalibacterium*, which have shown the strongest associations with diet and human health across studies. Both taxa can ferment dietary fibers (12–14) and are associated with plant-based foods (15, 16). *Prevotella* has been associated with both beneficial and detrimental health effects. For example, while *Prevotella* is associated with high-fiber diets (14), it has also been associated with inflammatory conditions, such as periodontal disease, rheumatoid arthritis, and metabolic syndrome (17–19). *Faecalibacterium*, by contrast, has consistently been associated with beneficial health effects (20). The strain-level contributions to these associations are not yet fully understood, but growing studies have revealed that *Prevotella* and *Faecalibacterium* are richer in strain diversity than previously appreciated (21, 22). A recent study showed that *Faecalibacterium* strain diversity can vary among people of different ages, populations, lifestyles, and disease statuses (23), with populations from less industrialized regions exhibiting higher prevalence and diversity. In our study, although individuals from Mexico did not exhibit markedly different *Prevotella* abundances at the genus level compared to those from the US (Fig. S2a), *Prevotella* strains displayed distinct, population-specific patterns (Fig. S2c). Conversely, while *Faecalibacterium* abundance varied across cohorts at the genus level (Fig. S2b), strain-level distributions were relatively consistent (Fig. S2d). These findings underscore the limitations of genus-level conclusions and highlight the importance of strain-level resolution when interpreting microbiome relationships.

Microbiome research has largely been limited to specific populations, which restricts generalizability. Our study takes a step toward addressing this gap; however, caution is warranted when interpreting findings from a minimal subset of the global population. Further research is needed to confirm these trends. Capturing a broader range of human and dietary diversity will help us untangle the complex relationships between microbial strains and diet. Our study indicates that specific associations with diet are detected only at the strain level. Efforts to develop a better understanding of whether other components of diet or the microbiome modify the relationships between a given strain and a given dietary item in a region-specific manner are warranted.

## Supplementary Material

Reviewer comments

## Data Availability

The data used in this study will be made available through the European Bioinformatics Institute (EBI) database (accession number: PRJEB11419) and Qiita Study #10317.

## SUPPLEMENTAL MATERIAL

Supplemental material msystems.00544-25-s0001.docx Supplemental methods, Table S1, and Figures S1 and S2.

OPEN PEER REVIEW reviewer-comments.pdf An accounting of the reviewer comments and feedback.

ASM does not own the copyrights to Supplemental Material that may be linked to, or accessed through, an article. The authors have granted ASM a non-exclusive, world-wide license to publish the Supplemental Material files. Please contact the corresponding author directly for reuse.

## References

[1] McDonald D, Hyde E, Debelius JW, Morton JT, Gonzalez A, Ackermann G, Aksenov AA, Behsaz B, Brennan C, Chen Y, et al.. 2018. American gut: an open platform for citizen science microbiome research. mSystems 3:e00031-18. doi:10.1128/mSystems.00031-18 29795809 PMC5954204

[2] Kristal AR, Kolar AS, Fisher JL, Plascak JJ, Stumbo PJ, Weiss R, Paskett ED. 2014. Evaluation of web-based, self-administered, graphical food frequency questionnaire. J Acad Nutr Diet 114:613–621. doi:10.1016/j.jand.2013.11.017 24462267 PMC3966309

[3] Cotillard A, Cartier-Meheust A, Litwin NS, Chaumont S, Saccareau M, Lejzerowicz F, Tap J, Koutnikova H, Lopez DG, McDonald D, Song SJ, Knight R, Derrien M, Veiga P. 2022. A posteriori dietary patterns better explain variations of the gut microbiome than individual markers in the American Gut Project. Am J Clin Nutr 115:432–443. doi:10.1093/ajcn/nqab332 34617562 PMC8827078

[4] Villarroel MBD, Jen A. 2019. Tables of summary health statistics for U.S. adults: 2018 national health interview survey. National Center for Health Statistics. Available from: http://www.cdc.gov/nchs/nhis/SHS/tables.htm

[5] (CDC) CfDCaP. 2017. National Health and Nutrition Examination Survey (NHANES). Hyattsville, MD. https://www.cdc.gov/nchs/nhanes/index.htm.

[6] Armstrong G, Martino C, Morris J, Khaleghi B, Kang J, DeReus J, Zhu Q, Roush D, McDonald D, Gonazlez A, Shaffer JP, Carpenter C, Estaki M, Wandro S, Eilert S, Akel A, Eno J, Curewitz K, Swafford AD, Moshiri N, Rosing T, Knight R. 2022. Swapping metagenomics preprocessing pipeline components offers speed and sensitivity increases. mSystems 7:e0137821. doi:10.1128/msystems.01378-21 35293792 PMC9040843

[7] Zhu Q, Huang S, Gonzalez A, McGrath I, McDonald D, Haiminen N, Armstrong G, Vázquez-Baeza Y, Yu J, Kuczynski J, et al.. 2022. Phylogeny-aware analysis of metagenome community ecology based on matched reference genomes while bypassing taxonomy. mSystems 7:e0016722. doi:10.1128/msystems.00167-22 35369727 PMC9040630

[8] Martino C, Morton JT, Marotz CA, Thompson LR, Tripathi A, Knight R, Zengler K. 2019. A novel sparse compositional technique reveals microbial perturbations. mSystems 4:e00016-19. doi:10.1128/mSystems.00016-19 30801021 PMC6372836

[9] Olm MR, Brown CT, Brooks B, Banfield JF. 2017. dRep: a tool for fast and accurate genomic comparisons that enables improved genome recovery from metagenomes through de-replication. ISME J 11:2864–2868. doi:10.1038/ismej.2017.126 28742071 PMC5702732

[10] Sanders JG, Sprockett DD, Li Y, Mjungu D, Lonsdorf EV, Ndjango J-B, Georgiev AV, Hart JA, Sanz CM, Morgan DB, Peeters M, Hahn BH, Moeller AH. 2023. Widespread extinctions of co-diversified primate gut bacterial symbionts from humans. Nat Microbiol 8:1039–1050. doi:10.1038/s41564-023-01388-w 37169918 PMC10860671

[11] Olm MR, Crits-Christoph A, Bouma-Gregson K, Firek BA, Morowitz MJ, Banfield JF. 2021. inStrain profiles population microdiversity from metagenomic data and sensitively detects shared microbial strains. Nat Biotechnol 39:727–736. doi:10.1038/s41587-020-00797-0 33462508 PMC9223867

[12] Benus RFJ, van der Werf TS, Welling GW, Judd PA, Taylor MA, Harmsen HJM, Whelan K. 2010. Association between Faecalibacterium prausnitzii and dietary fibre in colonic fermentation in healthy human subjects. Br J Nutr 104:693–700. doi:10.1017/S0007114510001030 20346190

[13] Kovatcheva-Datchary P, Nilsson A, Akrami R, Lee YS, De Vadder F, Arora T, Hallen A, Martens E, Björck I, Bäckhed F. 2015. Dietary fiber-induced improvement in glucose metabolism is associated with increased abundance of Prevotella. Cell Metab 22:971–982. doi:10.1016/j.cmet.2015.10.001 26552345

[14] Prasoodanan P K V, Sharma AK, Mahajan S, Dhakan DB, Maji A, Scaria J, Sharma VK. 2021. Western and non-western gut microbiomes reveal new roles of Prevotella in carbohydrate metabolism and mouth–gut axis. NPJ Biofilms Microbiomes 7:77. doi:10.1038/s41522-021-00248-x 34620880 PMC8497558

[15] De Filippis F, Pellegrini N, Laghi L, Gobbetti M, Ercolini D. 2016. Unusual sub-genus associations of faecal Prevotella and Bacteroides with specific dietary patterns. Microbiome 4:57. doi:10.1186/s40168-016-0202-1 27769291 PMC5073871

[16] Ahrens AP, Culpepper T, Saldivar B, Anton S, Stoll S, Handberg EM, Xu K, Pepine C, Triplett EW, Aggarwal M. 2021. A six-day, lifestyle-based immersion program mitigates cardiovascular risk factors and induces shifts in gut microbiota, specifically Lachnospiraceae, Ruminococcaceae, Faecalibacterium prausnitzii: a pilot study. Nutrients 13:3459. doi:10.3390/nu13103459 34684459 PMC8539164

[17] Nadkarni MA, Browne GV, Chhour K-L, Byun R, Nguyen K-A, Chapple CC, Jacques NA, Hunter N. 2012. Pattern of distribution of Prevotella species/phylotypes associated with healthy gingiva and periodontal disease. Eur J Clin Microbiol Infect Dis 31:2989–2999. doi:10.1007/s10096-012-1651-5 22684253

[18] Alpizar-Rodriguez D, Lesker TR, Gronow A, Gilbert B, Raemy E, Lamacchia C, Gabay C, Finckh A, Strowig T. 2019. Prevotella copri in individuals at risk for rheumatoid arthritis. Ann Rheum Dis 78:590–593. doi:10.1136/annrheumdis-2018-214514 30760471

[19] Guevara-Cruz M, Flores-López AG, Aguilar-López M, Sánchez-Tapia M, Medina-Vera I, Díaz D, Tovar AR, Torres N. 2019. Improvement of lipoprotein profile and metabolic endotoxemia by a lifestyle intervention that modifies the gut microbiota in subjects with metabolic syndrome. J Am Heart Assoc 8:e012401. doi:10.1161/JAHA.119.012401 31451009 PMC6755842

[20] Martín R, Rios-Covian D, Huillet E, Auger S, Khazaal S, Bermúdez-Humarán LG, Sokol H, Chatel J-M, Langella P. 2023. Faecalibacterium: a bacterial genus with promising human health applications. FEMS Microbiol Rev 47:fuad039. doi:10.1093/femsre/fuad039 37451743 PMC10410495

[21] Blanco-Míguez A, Gálvez EJC, Pasolli E, De Filippis F, Amend L, Huang KD, Manghi P, Lesker T-R, Riedel T, Cova L, Punčochář M, Thomas AM, Valles-Colomer M, Schober I, Hitch TCA, Clavel T, Berry SE, Davies R, Wolf J, Spector TD, Overmann J, Tett A, Ercolini D, Segata N, Strowig T. 2023. Extension of the Segatella copri complex to 13 species with distinct large extrachromosomal elements and associations with host conditions. Cell Host Microbe 31:1804–1819. doi:10.1016/j.chom.2023.09.013 37883976 PMC10635906

[22] Sakamoto M, Sakurai N, Tanno H, Iino T, Ohkuma M, Endo A. 2022. Genome-based, phenotypic and chemotaxonomic classification of Faecalibacterium strains: proposal of three novel species Faecalibacterium duncaniae sp. nov., Faecalibacterium hattorii sp. nov. and Faecalibacterium gallinarum sp. nov. Int J Syst Evol Microbiol 72:005379. doi:10.1099/ijsem.0.005379 35416766

[23] De Filippis F, Pasolli E, Ercolini D. 2020. Newly explored Faecalibacterium diversity is connected to age, lifestyle, geography, and disease. Curr Biol 30:4932–4943. doi:10.1016/j.cub.2020.09.063 33065016

