# Supplementary material for: A three-country analysis of the gut microbiome indicates taxon associations with diet vary by taxon resolution and population
Source: mSystems. 2025 Jun 30;10(7):e00544-25. doi: 10.1128/msystems.00544-25 (PMC12282181; doi:10.1128/msystems.00544-25)
Supplement: Supplemental material — Supplemental methods, Table S1, and Figures S1 and S2. [file msystems.00544-25-s0001.docx]

**Supplemental Methods**

Sample collection

Adult participants were consented through the Microsetta Initiative platform from the US, UK, and Mexico in accordance with University of California San Diego Human Research Protection Program (Protocol #: 141853) and the Instituto Nacional de Ciencias Medicas y Nutrición Salvador Zubiran (INCMNSZ) in Mexico (Protocol #: 3853). Fecal samples were collected using the Microsetta fecal collection kit (1). Specifically, swabs from used toilet paper were placed into tubes containing 2mL of 95% EtOH and stored at room temperature. In the US and UK, samples were shipped by FedEx directly to UC San Diego. In Mexico, samples were shipped within-country to INCMNSZ, which then shipped them to UC San Diego in aggregated batches at room temperature. On receipt, samples were checked into the Microsetta system. Broken samples, or samples which arrived without remaining EtOH were discarded. Subjects who submitted samples with incomplete information (e.g., an incomplete FFQ) were recontacted and assisted in completing the process. Only samples with complete information, including FFQ data, were used for sequencing and analysis.

Diet data collection and preparation

Participants completed the VioScreen™ food frequency questionnaire (FFQ) (Version 5, VioCare, Princeton, NJ) (2). The FFQ was adapted to reflect the dietary habits of each country. In short, participants reported their typical intake of a wide range of foods over the previous year, which allowed for a comprehensive assessment of dietary patterns and nutrient intake. The FFQ provided detailed estimates of macronutrient (e.g., carbohydrates, proteins, fats) and micronutrient (e.g., vitamins, minerals) intake, as well as a breakdown of food categories (e.g., fruits, vegetables, meats). In addition, *a priori* diet quality scores were calculated, including the Healthy Eating Index (HEI), which evaluates how well an individual’s diet aligns with dietary guidelines.

All food and beverage items in the Vioscreen FFQ were categorized according to the NOVA classification system (3), which divides foods into four categories based on the nature, purpose, and degree of industrial processing: 1) Unprocessed or minimally processed foods (e.g. fresh, squeezed, frozen, or dried fruit and vegetables, grains, legumes, nuts and seeds, meat, poultry, fish, eggs, milk and plain yogurt, coffee and tea) are foods altered by methods such as removal of inedible or unwanted parts, drying, powdering, squeezing, crushing, boiling, roasting, pasteurization, freezing, non-alcoholic fermentation, and other methods that do not add salt, sugar, oils or fats or other food substances to the original food, 2) Processed culinary ingredients (e.g. vegetable oils, butter, honey, sugar and, salt) are derived from foods or from nature by processes that include pressing, centrifuging, refining, extracting or mining, 3) Processed foods (e.g. canned vegetables and legumes in brine, fruit in syrup, salted or sugared nuts, salted and dried meats, canned fish, freshly made breads and cheeses and other foods made by adding salt, oil, sugar or other culinary ingredients), 4) Ultra-processed foods (e.g. carbonated soft drinks, sweet or savory packaged snacks, chocolate, candies, ice-cream, mass-produced packaged breads, margarines, cookies, pastries, breakfast cereals, flavored milk drinks, instant sauces, and pre-prepared ready-to-heat products) are formulations mostly made by ingredients not commonly used in culinary preparations such as high-fructose corn syrup, maltodextrin, hydrogenated oils, emulsifiers, colorings, flavor enhancers, artificial sweeteners, and other food additives added with the purpose to make the final product more hyper-palatable and attractive, and to prolong its shelf life.

Fruits and vegetables that have combined foods from different NOVA categories (n=4) specifically ‘unprocessed or minimally processed foods’ and ‘processed foods’ categories (e.g., ‘pineapple, fresh and canned’) were classified using the least degree of processing. Items that have combined foods including a food item from the ‘ultra-processed foods’ category (n=4) were classified using the higher degree of processing. Mexican traditional mixed dishes (e.g. enchiladas, chimichangas, flautas) were classified as “processed foods” for Mexico, since they are traditional dishes generally cooked at home with culinary ingredients, while in the United States and the United Kingdom they were classified as “ultra-processed foods” as usually they are industrial packaged ready-to-eat meals. For food items without a detailed description, we classified them according to the ingredient description from the Food and Nutrient Database for Dietary Studies (FNDDS). Vegetable soups were categorized as homemade if they did not specify that they were canned or instant.

DNA extraction and sequencing

DNA extraction was performed using the Matrix Method as described previously by Brennan et al (4). Briefly, swabbed samples were transferred to Matrix Tubes^TM^. 30 µL of 0.1, 0.5- and 1-mm zirconia-silica beads were added to each Matrix Tube using a LabTie bead dispenser (Molgen, Netherlands). 600 µL of Lysis buffer was added to each tube using The Biotek Multiflo bulk reagent dispenser (Agilent, CA, USA). The Matrix Tubes were capped using the Capit-All (ThermoFisher Scientific, MA, USA). Bead beating was performed on the SpexMiniG for 2 min at 1,200 x g. The remaining steps of the DNA extraction followed the manufacturer’s instructions with bead clean-ups performed using the automated KingFisher Flex Purification System (ThermoFisher Scientific, MA, USA). Extracted nucleic acids were stored at -80°C until further processing.

Sequencing

DNA was prepared for shotgun metagenomics sequencing as described previously (5). Metagenomic libraries were normalized by iSeq (Illumina) read count distribution to generate a final pool that made sequencing on the NovaSeq more efficient (6). The reads are available through the European Bioinformatics Institute (EBI) (accession number: PRJEB11419). The iSeq-normalized pools were run on a NovaSeq 6000 (Illumina, CA, USA). After sequencing, BCL files were demultiplexed, converted to per-sample-FASTQs, and quality controlled and human sequence filtered using the Qiita (7) admin plugin:<https://github.com/qiita-spots/qp-knight-lab-processing> following the recommendations from Sepich-Poore et al. (8). In summary, adapter trimming was done by fastp (9). Each sample was then filtered against each genome in the human pangenome (10), as well as T2T (11) and GRCh38 (12) via minimap2 (13) using two human genome references: GRCh38 with PhiX, and CHM13. The resulting per-sample-FASTQs were uploaded into Qiita study ID #10317 (<https://qiita.ucsd.edu/study/description/10317>; Prep IDs: 13358, 10967, 10903, 11594, 10915, 13280, 13279, 11595) and processed with its default processing workflow. The Metagenomic workflow applies a QC step using fastp and then applies woltka v0.1.4 (14), which uses the Bowtie2 aligner v2.3.2 (15) against the Web of Life release 2 (WoLr2) (16) database and automatically generates operational genomic unit (OGU) and functional profiles.

Metagenomic data processing

Starting with the OGU tables generated in Qiita, we removed samples with NA values in any of the following columns: level_of_wellbeing, antibiotic_history, host_age. We filtered out features not present in the Greengenes2 phylogeny (version 2022.10) (17) and then removed features with prevalence <10% at relative abundance < 0.0001%. Finally, we filtered out samples with fewer than 1,000,000 reads. The final table contained 1,215 samples and 1,783 features.

MAGs assembly, binning, and annotation

Metagenome Assembled Genomes (MAGs) were generated using a Snakemake (18) pipeline according to the protocol from Sanders et al (19). Trimmed and host-filtered sequences were assembled with megahit (20). From each country cohort, 5 prototypical samples were selected based on MinHash distances according to the method used in Zhu et al (14) and used for differential abundance binning using MaxBin2 (21), MetaBAT2 (22), and CONCOCT (23). Bins were refined with DASTool (24), then dereplicated using dRep (25), clustering at 95% average nucleotide identity (ANI). This yielded 728 High-quality and 363 Medium-quality draft genomes, per MIMAG standards (26). The pipeline used for assembly and binning can be found at (https://github.com/tanaes/sn-mg-pipeline/releases/tag/publication-thdmi). Final dereplicated MAGs were annotated using Bakta (27) and inStrain (28) using a second pipeline, which can be found at (<https://github.com/tanaes/snake_strainer/releases/tag/publication-thdmi>).

Statistical analysis

For the analysis of demographic variables between countries (Table 1), Kruskal-Wallis tests were performed for quantitative variables (i.e. age). For categorical variables, Chi-squared tests were performed. Metadata columns were normalized using zscores and averages by country were compared (Figure 1c). A PERMANOVA using every metadata column was conducted using the adonis2 R function(29). A random forest classifier using sklearn (30) was then used to predict cohort country across a 5-fold cross validation (Figure 1d).

After calculating nucleotide diversity for each species-level genome bins (sGB) within each sample using inStrain, individual diversity values were then pooled by bacterial genus to generate the data points displayed in Figure 2a. This analysis served as an exploratory approach to identify genera with high intra-genus variability potentially driven by dietary exposures to guide downstream strain-level analyses. Nucleotide diversity, when properly calculated, can provide a more robust and interpretable signal of ecological and evolutionary dynamics than abundance or prevalence at the genus level, particularly across diverse cohorts where compositional effects, sequencing depth variability, and sample processing differences may obscure underlying patterns (28; 30-31). Unlike relative abundance, which reflects community structure, nucleotide diversity captures within-taxon genetic heterogeneity, offering insights into microevolution, selective pressures, and strain-level turnover. Cross-cultural dietary differences, such as fiber-rich versus protein-rich diets, may not always shift the abundance of microbial genera, but can exert selective pressure that alters strain composition or genetic diversity within taxa. Thus, nucleotide diversity can help prioritize taxa likely to yield biologically meaningful insights in subsequent genome-resolved analyses. We note that nucleotide diversity was not used to infer function or strain identity, and although our estimates reflect genome-wide polymorphism across aligned regions, they do not resolve whether diversity is distributed evenly across the genome or concentrated in specific loci. To account for covariates and reduce dimensionality, lasso regression was utilized to test the correlation between the nucleotide diversity in species-level Genome Bins (sGB) and dietary variables (representing estimated micro and macronutrients) (Figure 2a). This approach applies L1 regularization to shrink less important coefficients to zero, effectively selecting a subset of relevant features while accounting for the influence of covariates. We used 5-fold cross-validation with the cv.glmnet() function in R to select the optimal regularization parameter (lambda) for LASSO regression (32-33). The final model was based on lambda.1se, the largest value of lambda within one standard error of the minimum cross-validated error, to favor simpler and more interpretable models. To account for potential confounding effects, we performed a sensitivity analysis by stratifying the dataset into subgroups based on key covariates (THDMI cohort, BMI, antibiotic history, and level of well-being) before applying LASSO regression. This allowed us to validate whether the selected variables remain significant across different subsets.

To assess the direction and significance of association between particular *Prevotella* or *Faecalibacterium* sGBs with dietary variables (Figure 2c), we first calculated the log ratio of each Prevotella or Faecalibacterium sGB’s abundance relative to the total abundance of all Prevotella or Faecalibacterium sGBs per sample. We then used linear mixed effects models in R’s lme4 package (34) to correlate the computed log ratio in a given sample to dietary variables of interest. We did this in two ways: first, on the whole cohort with the thdmi_cohort variable modeled as a random effect and second, on each country alone. In both cases, test statistics and p-values were calculated with the lmerTest package (35). Variation explained by covariates (BMI, antibiotic history, level of well-being) were accounted for, and the Benjamini-Hochberg correction was performed (with a 5% FDR) to account for multiple tests.

Using the WoLr2 OGU data (i.e., not MAGs), features were collapsed to the genus level. Missing values were imputed using a K-Nearest Neighbor alorithm. The Center Log-Ratio (CLR)-transformed values of *Prevotella* and *Faecalibacterium* to *Bacteroides* were then correlated to dietary variables using the lme4 package. THDMI cohort was modeled as a random effect, while BMI, antibiotic history, and level of well-being were modeled as fixed effects (as was each variable of interest). Significances were evaluated using lmerTest, and multiple comparisons were corrected using the Benjamini-Hochberg method with a 5% FDR. No dietary variables were significant correlated to any dietary variables after correcting for multiple comparison, except for the relationship between Prevotella and starch intake (Supplemental Figure 1a). The average log ratio of each of the 56 Prevotella MAGs to the sum of all Prevotella (Supplemental Figure 1b) and each of the 19 Faecalibacterium MAGs to the sum of all Faecalibacterium (Supplemental Figure 1c) across individuals in each cohort was compared.

The abundance of Prevotella and Faecalibacterium were then compared across cohorts using Kruskall-Wallis (Supplementary Figures 2a-b). Lastly, t-scores were computed to test the association between the nucleotide diversity in sGBs, pooled per individual by bacterial genus, and cohort, relative to Mexico (Supplementary Figure 2c).

Code used to analyze data will be made available at <https://github.com/lkhatib/3_Country_THDMI>.

STORMS Checklist can be found at <https://github.com/lkhatib/3_Country_THDMI/blob/main/STORMS_Excel_1.03.xlsx>

**Supplemental References**

1. Marotz C, Cavagnero KJ, Song SJ, McDonald D, Wandro S, Humphrey G, Bryant M, Ackermann G, Diaz E, Knight R. 2021. Evaluation of the effect of storage methods on fecal, saliva, and skin microbiome composition. Msystems 6:10.1128/msystems. 01329-20.

2. Kristal AR, Kolar AS, Fisher JL, Plascak JJ, Stumbo PJ, Weiss R, Paskett ED. 2014. Evaluation of web-based, self-administered, graphical food frequency questionnaire. Journal of the Academy of Nutrition and Dietetics 114:613-621.

3. Monteiro CA, Cannon G, Lawrence M, Costa Louzada Md, Pereira Machado P. 2019. Ultra-processed foods, diet quality, and health using the NOVA classification system. Rome: FAO 48.

4. Brennan C, Belda-Ferre P, Zuffa S, Charron-Lamoureux V, Mohanty I, Ackermann G, Allaband C, Ambre M, Boyer T, Bryant M. 2024. Clearing the plate: a strategic approach to mitigate well-to-well contamination in large-scale microbiome studies. mSystems:e00985-24.

5. Sanders JG, Nurk S, Salido RA, Minich J, Xu ZZ, Zhu Q, Martino C, Fedarko M, Arthur TD, Chen F. 2019. Optimizing sequencing protocols for leaderboard metagenomics by combining long and short reads. Genome biology 20:1-14.

6. Brennan C, Salido RA, Belda-Ferre P, Bryant M, Cowart C, Tiu MD, González A, McDonald D, Tribelhorn C, Zarrinpar A. 2023. Maximizing the potential of high-throughput next-generation sequencing through precise normalization based on read count distribution. Msystems 8:e00006-23.

7. Gonzalez A, Navas-Molina JA, Kosciolek T, McDonald D, Vázquez-Baeza Y, Ackermann G, DeReus J, Janssen S, Swafford AD, Orchanian SB. 2018. Qiita: rapid, web-enabled microbiome meta-analysis. Nature methods 15:796-798.

8. Sepich-Poore, G.D., McDonald, D., Kopylova, E., Guccione, C., Zhu, Q., Austin, G., Carpenter, C., Fraraccio, S., Wandro, S., Kosciolek, T. and Janssen, S. 2024. Robustness of cancer microbiome signals over a broad range of methodological variation. Oncogene 43:1127-1148.

9. Chen S, Zhou Y, Chen Y, Gu J. 2018. fastp: an ultra-fast all-in-one FASTQ preprocessor. Bioinformatics 34:i884-i890.

10. Liao WW, Asri M, Ebler J, et al. A draft human pangenome reference. Nature. 2023;617(7960):312–324. doi: 10.1038/s41586-023-05896-x

11. Rhie A, Nurk S, Cechova M, et al. The complete sequence of a human Y chromosome. Nature. 2023;621(7978):344–354. doi: 10.1038/s41586-023-06457-y

12. Schneider VA, Graves-Lindsay T, Howe K, et al. Evaluation of GRCh38 and de novo haploid genome assemblies demonstrates the enduring quality of the reference assembly. Genome Res. 2017;27(5):849–864. doi: 10.1101/gr.213611.116

13. Li H. 2018. Minimap2: pairwise alignment for nucleotide sequences. Bioinformatics 34:3094-3100.

14. Zhu Q, Huang S, Gonzalez A, McGrath I, McDonald D, Haiminen N, Armstrong G, Vázquez-Baeza Y, Yu J, Kuczynski J. 2022. Phylogeny-aware analysis of metagenome community ecology based on matched reference genomes while bypassing taxonomy. Msystems 7:e00167-22.

15. Langmead B, Salzberg, S. 2012. Fast gapped-read alignment with Bowtie 2, vol 9, p 357–359. *Nat Methods*.

16. Zhu Q, Mai U, Pfeiffer W, Janssen S, Asnicar F, Sanders JG, Belda-Ferre P, Al-Ghalith GA, Kopylova E, McDonald D. 2019. Phylogenomics of 10,575 genomes reveals evolutionary proximity between domains Bacteria and Archaea. Nature communications 10:5477.

17. McDonald D, Jiang Y, Balaban M, Cantrell K, Zhu Q, Gonzalez A, Morton JT, Nicolaou G, Parks DH, Karst S. 2022. Greengenes2 enables a shared data universe for microbiome studies. bioRxiv:2022.12. 19.520774.

18. Köster J, Rahmann S. 2012. Snakemake—a scalable bioinformatics workflow engine. Bioinformatics 28:2520-2522.

19. Sanders JG, Sprockett DD, Li Y, Mjungu D, Lonsdorf EV, Ndjango J-BN, Georgiev AV, Hart JA, Sanz CM, Morgan DB. 2023. Widespread extinctions of co-diversified primate gut bacterial symbionts from humans. Nature microbiology 8:1039-1050.

20. Li D, Liu C-M, Luo R, Sadakane K, Lam T-W. 2015. MEGAHIT: an ultra-fast single-node solution for large and complex metagenomics assembly via succinct de Bruijn graph. Bioinformatics 31:1674-1676.

21. Wu Y-W, Simmons BA, Singer SW. 2016. MaxBin 2.0: an automated binning algorithm to recover genomes from multiple metagenomic datasets. Bioinformatics 32:605-607.

22. Kang DD, Li F, Kirton E, Thomas A, Egan R, An H, Wang Z. 2019. MetaBAT 2: an adaptive binning algorithm for robust and efficient genome reconstruction from metagenome assemblies. PeerJ 7:e7359.

23. Alneberg J, Bjarnason BS, De Bruijn I, Schirmer M, Quick J, Ijaz UZ, Lahti L, Loman NJ, Andersson AF, Quince C. 2014. Binning metagenomic contigs by coverage and composition. Nature methods 11:1144-1146.

24. Sieber CM, Probst AJ, Sharrar A, Thomas BC, Hess M, Tringe SG, Banfield JF. 2018. Recovery of genomes from metagenomes via a dereplication, aggregation and scoring strategy. Nature microbiology 3:836-843.

25. Olm MR, Brown CT, Brooks B, Banfield JF. 2017. dRep: a tool for fast and accurate genomic comparisons that enables improved genome recovery from metagenomes through de-replication. The ISME journal 11:2864-2868.

26. Bowers RM, Kyrpides NC, Stepanauskas R, Harmon-Smith M, Doud D, Reddy T, Schulz F, Jarett J, Rivers AR, Eloe-Fadrosh EA. 2017. Minimum information about a single amplified genome (MISAG) and a metagenome-assembled genome (MIMAG) of bacteria and archaea. Nature biotechnology 35:725-731.

27. Schwengers O, Jelonek L, Dieckmann MA, Beyvers S, Blom J, Goesmann A. 2021. Bakta: rapid and standardized annotation of bacterial genomes via alignment-free sequence identification. Microbial genomics 7:000685.

28. Olm MR, Crits-Christoph A, Bouma-Gregson K, Firek BA, Morowitz MJ, Banfield JF. 2021. inStrain profiles population microdiversity from metagenomic data and sensitively detects shared microbial strains. Nature Biotechnology 39:727-736.

29. Oksanen J, Blanchet FG, Friendly M, Kindt R, Legendre P, McGlinn D, Minchin PR, O'Hara RB, Simpson GL, Solymos P, Stevens MHH, Szoecs E, Wagner H. 2022. vegan: Community Ecology Package. R package version 2.6-4. Available at: <https://CRAN.R-project.org/package=vegan>

30. Brito IL, Alm EJ. 2016. Tracking strains in the microbiome: insights from metagenomics and models. Front Microbiol 7:712.

31. Pasolli E, Asnicar F, Manara S, Zolfo M, Karcher N, Armanini F, Beghini F, Manghi P, Tett A, Ghensi P, Collado MC, Rice BL, DuLong C, Morgan XC, Golden CD, Quince C, Huttenhower C, Segata N. 2019. Extensive unexplored human microbiome diversity revealed by over 150,000 genomes from metagenomes spanning age, geography, and lifestyle. Cell 176:649–662.e20.

32. Pedregosa F, Varoquaux G, Gramfort A, Michel V, Thirion B, Grisel O, Blondel M, Prettenhofer P, Weiss R, Dubourg V, Vanderplas J, Passos A, Cournapeau D, Brucher M, Perrot M, Duchesnay É. 2011. Scikit-learn: Machine learning in Python. Journal of Machine Learning Research 12:2825-2830.

33. Friedman, J.H., Hastie, T. and Tibshirani, R., 2010. Regularization paths for generalized linear models via coordinate descent. Journal of statistical software, 33: 1-22.

32. Simon, N., Friedman, J.H., Hastie, T. and Tibshirani, R., 2011. Regularization paths for Cox's proportional hazards model via coordinate descent. Journal of statistical software, 39: 1-13.

34. Bates D. 2014. Fitting linear mixed-effects models using lme4. arXiv preprint arXiv:14065823.

35. Kuznetsova A, Brockhoff PB, Christensen RHB. 2017. lmerTest package: tests in linear mixed effects models. Journal of statistical software 82

**Table S1.** Dietary variables that were tested for significant associations with each Prevotella and Faecalibacterium sGB by country.

| Amount_Alcohol_in_g | Hei2015_Added Sugars | Percent_of_calories_from_Added_Sugar |
| --- | --- | --- |
| Amount_Animal_Protein_in_g | Hei2015_Dairy | Percent_of_calories_from_Alcohol |
| Amount_Insoluble_Dietary_Fiber_in_g | Hei2015_Fatty Acids | Percent_of_calories_from_Carbohydrate |
| Amount_Pectins_in_g' | Hei2015_Greens and Beans | Percent_of_calories_from_Fat |
| Amount_Soluble_Dietary_Fiber_in_g | Hei2015_Refined Grains | Percent_of_calories_from_Monounsaturated_Fat |
| Amount_Starch_in_g | Hei2015_Saturated Fat | Percent_of_calories_from_Polyunsaturated_Fat |
| Amount_Total_Carbohydrate_in_g | Hei2015_Seafood and Plant Proteins | Percent_of_calories_from_Protein |
| Amount_Total_Dietary_Fiber_in_g | Hei2015_Sodium | Percent_of_calories_from_Saturated_Fat |
| Amount_Total_Fat_in_g | Hei2015_Total Fruit | Processed_Calories_Nova_processed_foods_per1000kcal |
| Amount_Total_Protein_in_g | Hei2015_Total HEI Score | Processed_Calories_Nova_ultra_processed_foods_per1000kcal |
| Amount_Total_Sugars_in_g | Hei2015_Total Protein Foods | Processed_Calories_Nova_unprocessed_or_minimally_processed_foods_per1000kcal |
| Amount_Vegetable_Protein_in_g | Hei2015_Total Vegetables | Types_of_plants_coded |
|  | Hei2015_Whole Fruit |  |
|  | Hei2015_Whole Grains |  |

**Supplemental Figures**

­­­­­
